# Protein-energy malnutrition and worse outcomes after major cancer surgery: A nationwide analysis

**DOI:** 10.3389/fonc.2023.970187

**Published:** 2023-01-17

**Authors:** Jiewen Jin, Xianying Zhu, Zhantao Deng, Pengyuan Zhang, Ying Xiao, Hedong Han, Yanbing Li, Hai Li

**Affiliations:** ^1^ Department of Endocrinology, The First Affiliated Hospital, Sun Yat-Sen University, Guangzhou, China; ^2^ Department of Intensive Care Unit, Sun Yat-sen University Cancer Center, State Key Laboratory of Oncology in South China, Collaborative Innovation Center for Cancer Medicine, Guangzhou, China; ^3^ Department of Orthopedics, Guangdong Provincial People's Hospital (Guangdong Academy of Medical Sciences), Southern Medical University, Guangzhou, China; ^4^ Department of Respiratory and Critical Care Medicine, Jinling Hospital, Nanjing University School of Medicine, Nanjing, China

**Keywords:** protein-energy malnutrition (PEM), major cancer surgery, mortality, postoperative complications, nationwide analysis

## Abstract

**Background:**

Protein-energy malnutrition (PEM) has been recognized as a poor prognostic factor in many clinical issues. However, nationwide population studies concerning the impact of PEM on outcomes after major cancer surgery (MCS) are lacking. We aimed to evaluate the postoperative outcomes associated with PEM following MCS.

**Methods:**

By using the Nationwide Inpatient Sample database, data of patients undergoing MCS including colectomy, cystectomy, esophagectomy, gastrectomy, hysterectomy, lung resection, pancreatectomy, or prostatectomy were analyzed retrospectively from 2009 to 2015, resulting in a weighted estimate of 1,335,681 patients. The prevalence trend of PEM, as well as mortality and major complications after MCS were calculated. Multivariable regression analysis was applied to estimate the impact of PEM on postoperative outcomes after MCS.

**Results:**

PEM showed an estimated annual percentage increase of 7.17% (95% confidence interval (CI): 4-10.44%) from 2009 to 2015, which contrasts with a 4.52% (95% CI: -6.58–2.41%) and 1.21% (95% CI: -1.85–0.56%) annual decrease in mortality and major complications in patients with PEM after MCS. PEM was associated with increased risk of mortality (odds ratio (OR)=2.26; 95% CI: 2.08-2.44; P < 0.0001), major complications (OR=2.46; 95% CI: 2.36-2.56; P < 0.0001), higher total cost ($35814 [$22292, $59579] vs. $16825 [$11393, $24164], P < 0.0001), and longer length of stay (14 [9-21] days vs. 4 [2-7] days, P < 0.0001), especially in patients underwent prostatectomy, hysterectomy and lung resection.

**Conclusions:**

PEM was associated with increased worse outcomes after major cancer surgery. Early identification and timely medical treatment of PEM for patients with cancer are crucial for improving postoperative outcomes.

## 1 Introduction

Protein-energy malnutrition (PEM), caused by depleted energy and nutrient stores, often leads to alterations in body weight and composition and compromised functioning (1). PEM has been recognized as a poor prognostic factor in many clinical issues, such as acute myocardial infarction, sepsis, and heart failure (1–3). Consequently, the importance of identification and management of PEM has been highlighted in recent years.

Cancer is a major public health problem worldwide and has been the second leading cause of death in the United States (4). In China, cancer death accounted for 24% of all-cause of death during 2014 to 2018 and is the leading cause of death in the population less than 65 years old (5). Metabolic diseases, such as obesity and diabetes, are vital risk factors for cancers, which may resulted from energy imbalance and inflammation (6, 7). Patients with cancer are at a particularly high risk of malnutrition. The etiology is complicated, including impaired food intake due to host and therapeutic factors, increased energy and protein demands, and metabolic abnormalities (8). Although there is a relatively high prevalence of malnutrition ranging from 20% to more than 70% in patients with cancer, only 30-60% of those at risk of malnutrition received nutritional support (8).

Surgery, one of the major cancer treatments, can negatively regulate nutrition status due to the catabolic impact of the surgery itself, inflammation induction, and enhanced metabolic stress response (9). Malnutrition is associated with negative clinical outcomes following certain cancer surgeries such as esophagectomy, gastrectomy, colectomy, hepatectomy, pancreatectomy, lung resection, cystectomy, and hysterectomy (10–16). However, nationwide population studies on the impact of PEM on outcomes after major cancer surgery (MCS) are lacking.

Therefore, we used National Inpatient Sample (NIS) database to explore: 1) prevalence and temporal trends of PEM who underwent MCS; 2) the impact of PEM on mortality, major complications, total cost, and length of stay (LOS) after MCS; 3) the influence of surgical type on the perioperative outcomes of PEM patients.

## 2 Methods

### 2.1 Data source and study population

It is a retrospective cohort study investigating the influence of PEM on perioperative outcomes in patients undergoing MCS. Patients aged 18-90 years old who were admitted between January 1^st^, 2009 to December 31^st^, 2015 and primarily for MCS were included from NIS database. The NIS database is the largest all-payer administrative database that includes a 20% stratified sample of United States inpatient hospitalizations from nonfederal community hospitals (17). The NIS database provides information including patient features, primary diagnosis, up to 29 secondary diagnoses, up to 15 inpatient procedures, hospitalization costs, and LOS (1). We selected a total of eight major surgical oncological procedures (colectomy, cystectomy, esophagectomy, gastrectomy, hysterectomy, lung resection, pancreatectomy, and prostatectomy) as MCS and evaluated their perioperative outcomes. Relying on specific International Classification of Disease, Ninth Revision, Clinical Modification (ICD-9-CM) procedure codes, each surgical procedure was assessed independently, and analyses were restricted to cancer diagnoses only (**Supplementary Table 1**) (18).

### 2.2 Ethical approval

The data collected in the present study is from an open access database, where the ethics approval and consent to participated had been made when the database setup. Hence, it is not applicable in the present study.

### 2.3 Outcomes

The primary outcome was perioperative outcomes, which included mortality, major complications, total costs, and LOS. Mortality, total costs, and LOS were directly extracted from NIS database. Major complications were identified through ICD-9-CM diagnosis codes, defined as pneumonia, pulmonary embolism, renal failure, acute ischemic stroke, acute myocardial infarction, cardiac arrest, adult respiratory distress syndrome, sepsis, and septic shock (**Supplementary Table 2**).

### 2.4 Predictor

PEM (primary predictor) was identified with ICD-9-CM diagnosis codes (260, 261, 262, 263, 2698, 7994, 7833, 7837, 78321, 78322), which included kwashiorkor, marasmus, cachexia, other severe protein-calorie malnutrition (severe and unspecified), adult failure to thrive, loss of weight, and underweight. These set of diagnosis codes is recommended by the Academy of Nutrition and Dietetics, and the American Society for Parenteral and Enteral Nutrition and have been used by many studies (1–3, 19).

### 2.5 Patient and hospital characteristics

For all patients, the following independent variables were potential confounders and were available for analyses: patient age at hospitalization, sex, elective status, race (white, black, Hispanic, other (Asian, Pacific Islander, or Native American), or unknown), insurance status, income quartile, hospital type, hospital region, hospital bed size, baseline comorbidities, and type of cancer surgery. All the potential confounders were identified either as already present in NIS database or clinical classification software codes to abstract them from the diagnosis variables (2).

Patient age was regarded as a continuous variable. Insurance categories included Medicare, Medicaid, private insurance, and other insurance types (self-pay). Income was stratified into four quartiles based on the average annual household income of the zip code of residence (0-25^th^, 26-50^th^, 51-75^th^, and 76-100^th^ quartiles). The hospital type was categorized by the hospital’s teaching status (rural, urban non-teaching, and teaching). The Hospital region included the Northeast, Midwest, South, and West regions. Hospitals bed size was stratified as small, medium and large hospital size (20). Baseline comorbidities were quantified using an Elixhauser comorbidity index (ECI) (21). Elixhauser comorbid conditions included: alcohol abuse, acquired immune deficiency syndrome, deficiency anemias, rheumatoid arthritis/collagen vascular diseases, chronic blood loss anemia, congestive heart failure, chronic pulmonary disease, coagulopathy, depression, diabetes without complications, diabetes with chronic complications, drug abuse, hypertension (uncomplicated and complicated), hypothyroidism, liver disease, lymphoma, fluid and electrolyte disorders, obesity, other neurological disorders, paralysis, peripheral vascular disorders, psychoses, pulmonary circulation disorders, renal failure, peptic ulcer disease excluding bleeding, valvular disease, and weight loss (**Supplementary Table 3**).

### 2.6 Statistical analysis

All analyses were performed on the provided NIS population (268,595 individuals), and P-values were calculated for the weighted population (1,335,681 individuals). Descriptive statistics were generated on frequencies and proportions of categorical variables (gender, type of admission, race, insurance status, median zip code household income, hospital teaching status, hospital region, hospital bed size, ECI, and type of cancer surgery) and stratified according to PEM occurrence. Means were reported for continuously coded variables (age). Chi-square tests were applied to compare the statistical significance of differences within categorical variables. Temporal trends in rates were analyzed by the estimated annual percent change (EAPC) using linear regression analyses. To further investigate the relationship between PEM and outcomes after MCS, we used multivariable logistic regression models adjusted for age, sex, race, type of insurance, elective status, income quartile, hospital type, hospital region, hospital bed size, ECI, and surgical type. Subgroup analyses stratified by surgical type were applied. Sensitivity analyses were performed to test the robustness of our findings. We reassessed the relationship between PEM and clinical outcomes in patients undergoing MCS based on a double robust inverse probability of treatment weighting method (22). The probability of treatment or propensity score was calculated using multivariable logistic regression models adjusted for the aforementioned variables. All statistical analyses were performed using SAS software version 9.4 (SAS Institute, Cary, NC). Statistical significance was defined as a P-value < 0.05 on two-tailed testing.

## 3 Results

### 3.1 Baseline descriptive statistics

A total of 268,595 (weighted 1,335,681) patients who underwent MCS were selected from 2009 to 2015 of NIS database. Among them, 7.1% of patients had PEM. Patient with PEM were older, more likely to be female, higher percentage of black subjects, more likely to have Medicare as their primary health insurance and a lower income (**Table 1**). It was not surprising that patients with PEM had a higher comorbidity burden with a greater proportion of patients with ECI ≥ 3 (77.39% vs. 28.78%, P < 0.0001) (**Table 1**). As shown in **Supplementary Table 3**, almost all of the Elixhauser comorbid conditions were statistically significant between patients who underwent MCS with and without PEM (P < 0.05 for all).

**Table 1:** Baseline characteristics in patients undergoing major cancer surgery with and without PEM.

Variables	With PEM (N=19201, %)	Without PEM (N=249394, %)	P-value
**Mean age (SE)**	69.67(0.12)	64.92(0.06)	<0.0001
**Female**	8831(45.99)	94160(37.76)	<0.0001
**Elective admission**	10250(53.38)	213499(85.61)	<0.0001
**Race**			<0.0001
White	13051(67.97)	173797(69.69)	
Black	2251(11.72)	24716(9.91)	
Hispanic	1109(5.78)	14459(5.80)	
Other	1061(5.53)	13353(5.35)	
Unknown	1729(9.00)	23069(9.25)	
**Type of insurance**			<0.0001
Medicare	12542(65.32)	120503(48.32)	
Medicaid	1412(7.35)	12054(4.83)	
Private	4239(22.08)	105314(42.23)	
Others	1008(5.25)	11523(4.62)	
**Income quartile**			<0.0001
0-25th	5551(28.91)	56643(22.71)	
26-50th	5031(26.20)	61041(24.48)	
51-75th	4556(23.73)	62358(25.00)	
76-100th	4063(21.16)	69352(27.81)	
**Hospital type**			<0.0001
Rural	1507(7.85)	15244(6.11)	
Urban non-teaching	5711(29.74)	66993(26.86)	
Urban teaching	11983(62.41)	167157(67.03)	
**Hospital region**			0.0002
Northeast	3229(16.82)	51167(20.52)	
Midwest	5088(26.50)	60821(24.39)	
South	7238(37.70)	89298(35.81)	
West	3646(18.99)	48108(19.29)	
**Hospital bed size**			0.0247
Small	2040(10.62)	29171(11.70)	
Medium	4525(23.57)	54208(21.74)	
Large	12636(65.81)	166015(66.57)	
**ECI**			<0.0001
0	41(0.21)	52898(21.21)	
1	1259(6.56)	69003(27.67)	
2	3042(15.84)	55719(22.34)	
≥3	14859(77.39)	71774(28.78)	
**Cancer surgical type**			<0.0001
Colectomy	9877(51.44)	55450(22.23)	
Cystectomy	1185(6.17)	11112(4.46)	
Esophagectomy	743(3.87)	2272(0.91)	
Gastrectomy	1803(9.39)	6367(2.55)	
Lung resection	2308(12.02)	43950(17.62)	
Hysterectomy	511(2.66)	33048(13.25)	
Pancreatectomy	2586(13.47)	10513(4.22)	
Prostatectomy	188(0.98)	86682(34.76)	

SE, standard error; ECI, Elixhauser comorbidity index; PEM, protein-energy malnutrition.

**Figure 1:**
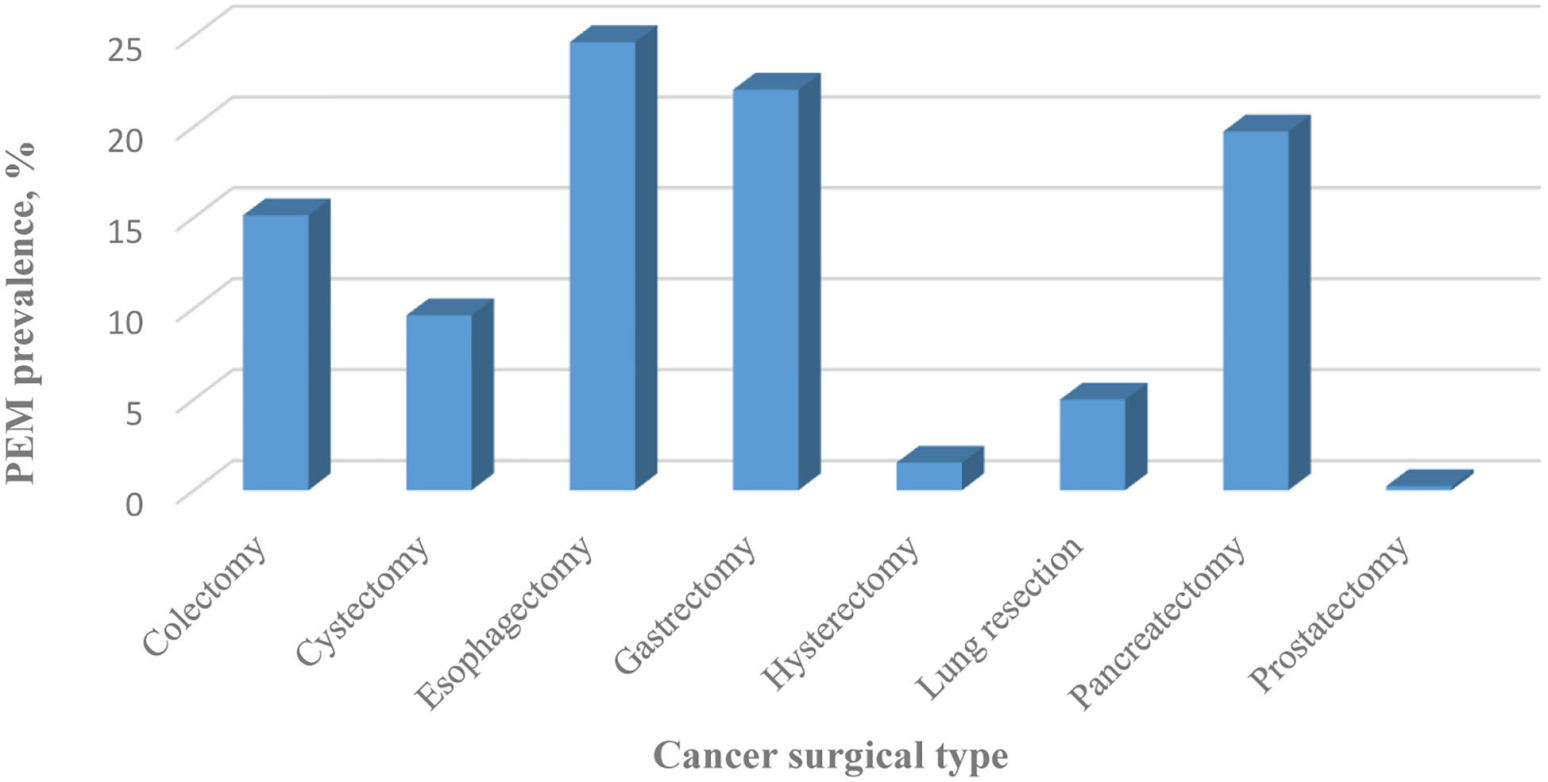
Prevalence of PEM classified by cancer surgery type between 2009 and 2015 in the United States.

Concerning the type and admission of surgery, patients with PEM had lower proportion of elective admission (53.38% vs. 85.61%, P < 0.0001) with highest proportion of colectomy (51.44%), followed by pancreatectomy (13.47%), lung resection (12.02%), gastrectomy (9.39%), cystectomy (6.17%), esophagectomy (3.87%), hysterectomy (2.66%) and prostatectomy (0.98%) (**Table 1**). Patients who underwent operations for gastrointestinal (GI) cancers had the highest prevalence of PEM. Esophageal cancer ranked first (24.6502%), gastric cancer ranked second (22.029%), followed by pancreatic cancer (19.7319%), and colon cancer (15.1097%). Patients treated surgically for lung cancer (4.9766%) and bladder cancer (9.6109%) had moderate rates of PEM. Patients who underwent operations for uterine cancer (1.5188%) and prostate cancer (0.2171%) had the lowest rates of PEM (**Figure 1**).

### 3.2 Temporal trends of PEM, mortality and major complications

**Figure 2:**
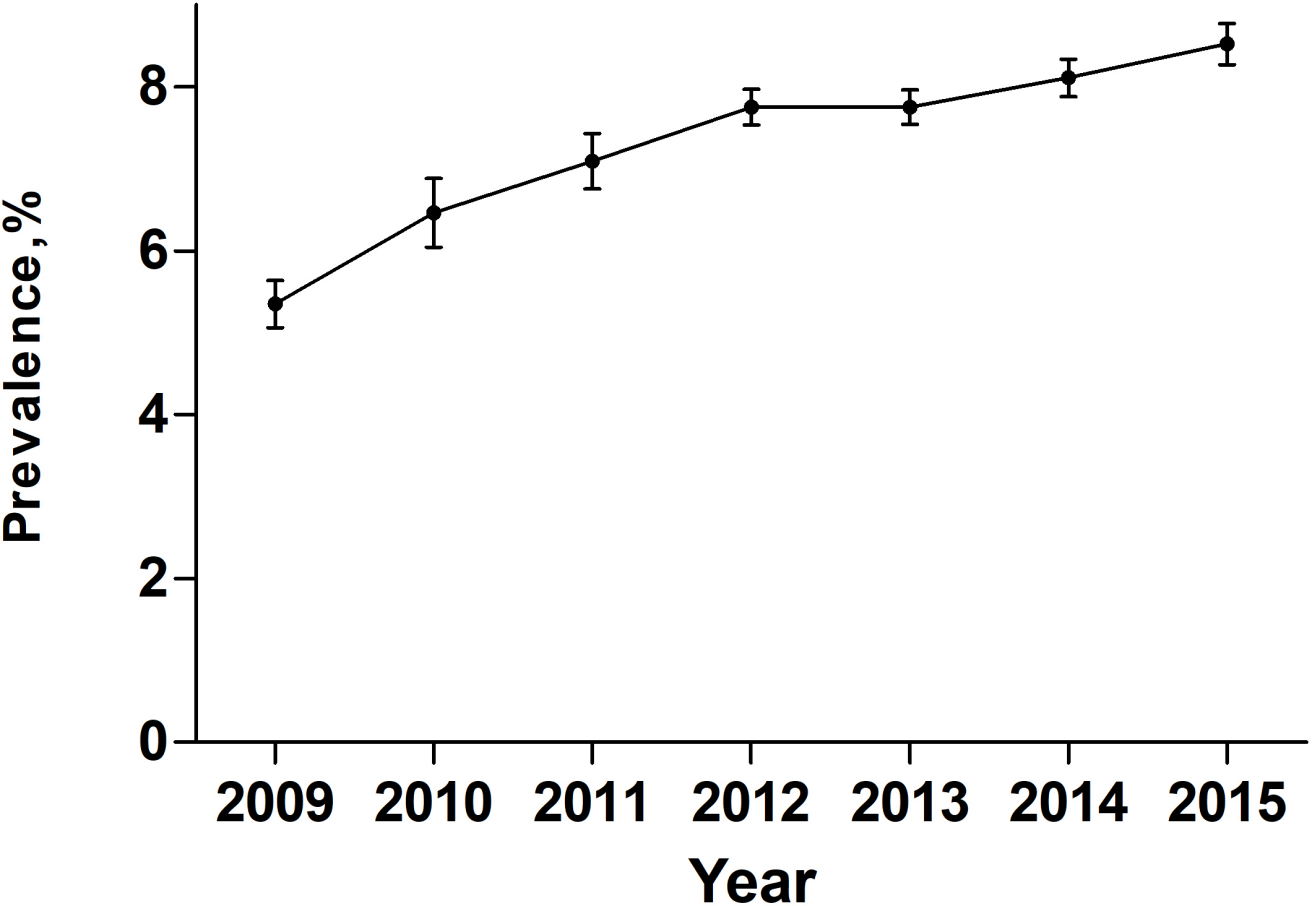
Prevalence of PEM in patients undergoing major cancer surgery patients between 2009 and 2015 in the United States.

**Figure 3:**
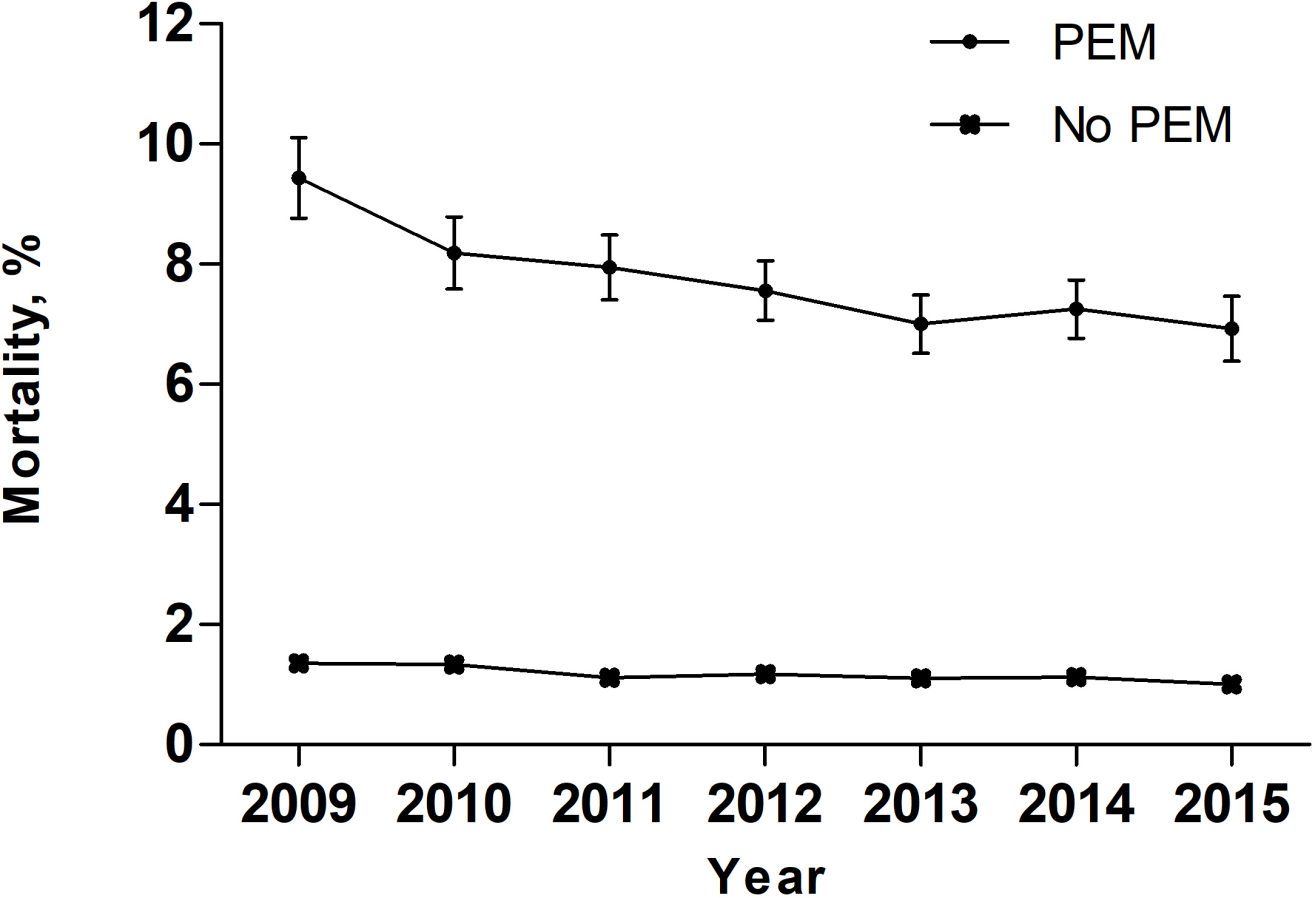
Mortality in patients with and without PEM between 2009 and 2015 in the United States.

**Figure 4:**
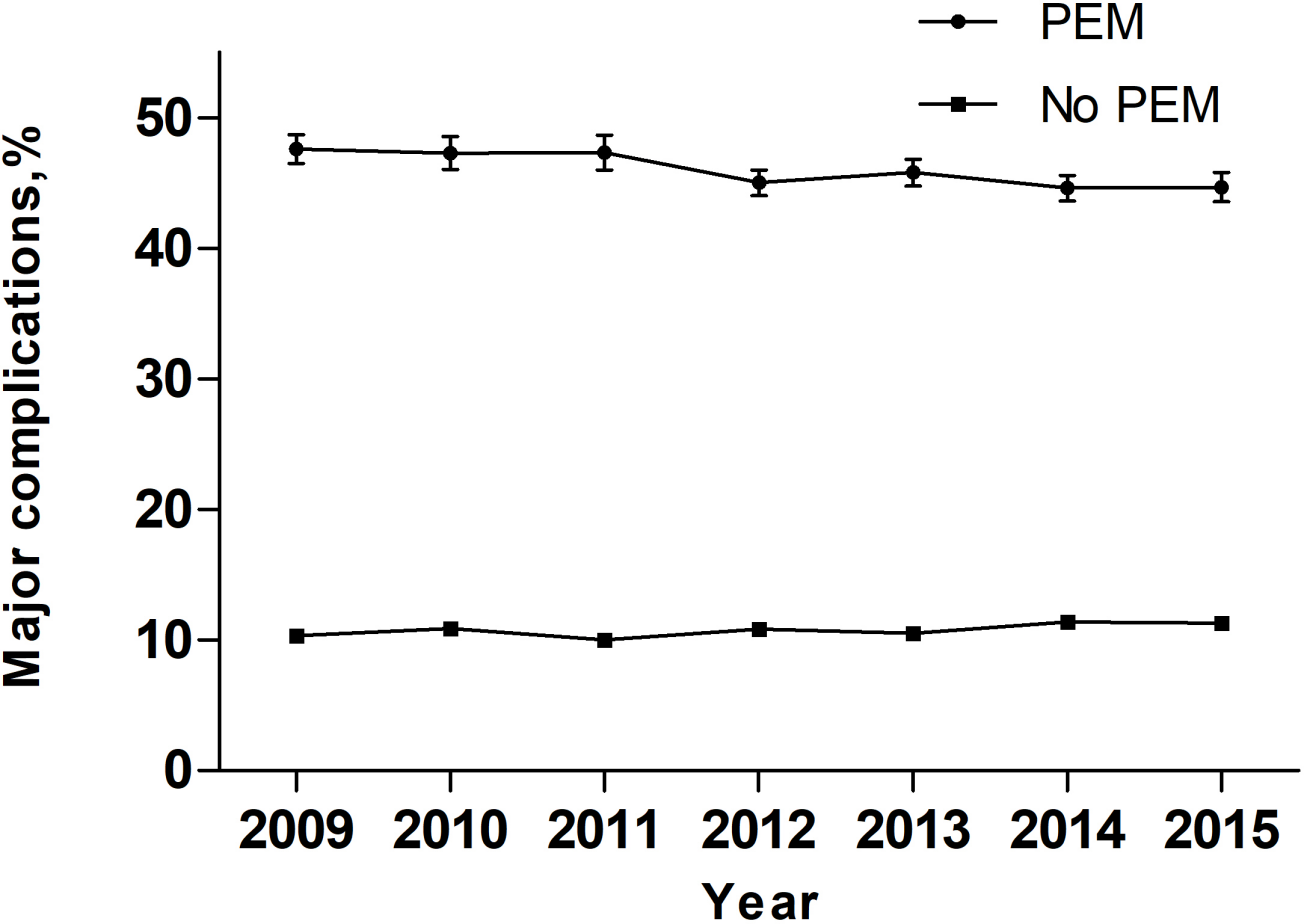
Major complications in patients with and without PEM between 2009 and 2015 in the United States.

Over the entire study period, temporal trend analyses showed that the EAPC of PEM was +7.17% (95% confidence interval [CI]: 4-10.44; P = 0.0019) (**Figure 2**). During the same period, the EAPC of mortality in patients with PEM was -4.52% (95% CI: -6.58–2.41, P < 0.01) while the EAPC of mortality in patients without PEM was -4.21% (95% CI: -6.68–1.68, P < 0.01) (**Figure 3**). Meanwhile, the EAPC of major complications in patients with PEM was -1.21% (95% CI: -1.85–0.56, P = 0.0048), and the EAPC of major complications in patients without PEM showed no significant change (EAPC = 1.45, 95% CI: -0.43-3.36, P = 0.1046) (**Figure 4**).

### 3.3 Perioperative outcomes after MCS in patient with PEM

Patients with PEM had poorer perioperative outcomes after MCS. The mortality rate was 7.77% in patients with PEM, which was 2.26-fold higher than those without PEM (1.19%) (odds ratio [OR]= 2.26, 95% CI: 2.08-2.44, P<0.0001) (**Table 2**). Moreover, PEM was associated with higher total cost ($35814 vs. $16825, P < 0.0001) and longer LOS (14 days vs. 4 days, P < 0.0001) (**Table 2**).

**Table 2:** Comparison of clinical outcomes following major cancer surgery in patients with and without PEM.

Outcomes	Event rates (%)		Adjusted OR (95%CI)^#^	P-value
	With PEM	Without PEM		
Mortality	1491(7.77)	2960(1.19)	2.26(2.08,2.44)	<0.0001
Major complications	8850(46.09)	26671(10.69)	2.46(2.36,2.56)	<0.0001
Pneumonia	4155(21.64)	11307(4.53)	2.52(2.40,2.65)	<0.0001
Pulmonary embolism	541(2.82)	1523(0.61)	1.62(1.44,1.82)	<0.0001
Renal failure	4398(22.91)	12131(4.86)	1.98(1.89,2.07)	<0.0001
Acute ischemic stroke	246(1.28)	662(0.27)	1.98(1.68,2.33)	<0.0001
Acute myocardial infarction	446(2.32)	1482(0.59)	1.44(1.28,1.62)	<0.0001
Cardiac arrest	283(1.47)	822(0.33)	1.88(1.61,2.20)	<0.0001
Adult respiratory distress syndrome	2733(14.23)	5784(2.32)	2.51(2.36,2.68)	<0.0001
Sepsis	1272(6.62)	1895(0.76)	3.08(2.82,3.36)	<0.0001
Septic shock	2002(10.43)	2590(1.04)	3.55(3.28,3.86)	<0.0001
Mechanical Ventilation	3698(19.26)	8125(3.26)	2.62(2.47,2.77)	<0.0001
Total cost, median (IQR)	35814(22292, 59579)	16825(11393, 24164)	0.39	<0.0001
Length of stay, median (IQR)	14(9,21)	4(2,7)	0.44	<0.0001

PEM, protein-energy malnutrition; IQR, interquartile range; OR, odds ratio; CI, confidence interval.

^#^Adjusted for age, sex, race, type of insurance, elective status, income quartile, hospital type, hospital region, hospital bed size, Elixhauser comorbidity index and surgical type.

Considering major complications, PEM group showed a 2.46-fold increase of risk when compared with non-PEM group (OR=2.46, 95% CI: 2.36-2.56, P < 0.0001) (**Table 2**). More specifically, renal failure (22.91%), pneumonia (21.64%), adult respiratory distress syndrome (14.23%), and septic shock (10.43%) were most common in the PEM group. When compared with non-PEM group, patients with PEM had higher risk of septic shock (OR=3.55, 95%CI: 3.28-3.86) and sepsis (OR=3.08, 95%CI: 2.82-3.36), followed by pneumonia (OR=2.52, 95%CI: 2.40-2.65), adult respiratory distress syndrome (OR= 2.51, 95%CI: 2.36-2.68), renal failure (OR=1.98, 95%CI: 1.89-2.07), acute ischemic stroke (OR=1.98, 95%CI: 1.68-2.33), cardiac arrest (OR=1.88, 95%CI: 1.61-2.20), pulmonary embolism (OR=1.62, 95%CI: 1.44-1.82) and acute myocardial infarction (OR=1.44, 95%CI: 1.28-1.62). Moreover, patients with PEM showed a 2.62-fold increase in the need for mechanical ventilation after MCS compared with patients without PEM (OR=2.62, 95%CI: 2.47-2.77, P < 0.0001) (**Table 2**).

### 3.4 The influence of surgical type on perioperative outcomes

In order to investigate the influence of surgical type on the perioperative outcomes of PEM patients, subgroup analysis was conducted. The rate of mortality varied among surgical types (**Supplementary Table 4**). PEM patients underwent lung resection (10.27%) and colectomy (8.35%) had the highest mortality rate while those underwent prostatectomy had the lowest mortality (1.6%).

The risk of mortality and major complications also varied among surgical types. Patients with PEM underwent prostatectomy had the highest risk of mortality (OR=13.59, 95%CI: 3.26-56.65), and major complications (OR=7.34, 95%CI: 5.18-10.38), followed by patients underwent hysterectomy (mortality: OR=9.81; major complications, OR=5.38) and lung resection (mortality: OR=4.64; major complications, OR=3.49), which were all non-GI operations (**Table 3**). On the other hand, gastrointestinal operations, such as colectomy, esophagectomy, gastrectomy, pancreatectomy and cystectomy, had relatively lower risk (1-2 folds) of perioperative mortality in patients with PEM (**Table 3**).

**Table 3:** Subgroup analysis according to cancer surgical type.

Surgical type	Mortality	Major complications	Total cost	LOS
	OR(95%CI)^#^	OR(95%CI)^#^	Coefficient^#^	Coefficient^#^
Colectomy	2.05(1.86,2.26)	2.34(2.22,2.46)	0.39	0.38
Cystectomy	2.08(1.46,2.97)	3.04(2.64,3.51)	0.41	0.56
Esophagectomy	1.48(0.98,2.22)	1.86(1.52,2.27)	0.25	0.29
Gastrectomy	1.83(1.42,2.37)	2.01(1.77,2.28)	0.29	0.32
Hysterectomy	9.81(6.05,15.93)	5.38(4.36,6.63)	0.74	1.16
Lung resection	4.64(3.89,5.53)	3.49(3.17,3.84)	0.51	0.59
Pancreatectomy	1.51(1.21,1.87)	1.96(1.76,2.19)	0.29	0.37
Prostatectomy	13.59(3.26,56.65)	7.34(5.18,10.38)	0.65	1.14

OR, odds ratio; LOS, length of stay; CI, confidence interval.

^#^Adjusted for age, sex, race, type of insurance, elective status, income quartile, hospital type, hospital region, hospital bed size and Elixhauser comorbidity index.

### 3.5 Sensitivity analysis

In order to eliminate the influence of residual confounders on the robustness of the results, a sensitivity analysis was conducted. All the results, including mortality, major complications, total costs, and LOS remained statistically significant after the double robust inverse probability of treatment weighting method (**Supplementary Table 5**).

## 4 Discussion

In the present study, we found the rate of PEM in patients underwent MSC was 7.1% by analyzing data of more than 1 million patients from NIS database. Patients with PEM were older, more likely to be female, higher percentage of black subjects, a lower income, lower proportion of elective admission and higher proportion of operations for GI cancers. The EAPC of PEM was +7.17%. PEM patients had higher risk of mortality and major complications, as well as higher total cost and longer LOS when compared with non-PEM patients after MCS. PEM patients who underwent lung resection and colectomy had the highest mortality rate while PEM significantly increased the risk of mortality and major complications in those underwent prostatectomy, hysterectomy and lung resection.

PEM is a common problem in cancer patients and has been recognized as a poor prognostic factor of postoperative complications and death (23). In the past decade, early identification and prevention of PEM have attracted increasing attention, many screening tools for malnutrition and guidelines for clinical nutrition in cancer have been advanced (24). In the present study, we reported that the prevalence of PEM in patients undergoing MCS was 7.1% (**Table 1**), which is much lower than other reports to focus on the prevalence of malnutrition in patients with cancer (20-70%) (8). The inconsistency of PEM prevalence was contributing to difference of cancer stage, cancer type and patient age (25). It is reported that the prevalence of moderate and severe malnutrition in stage III and IV patients was 79%, which is significantly higher than in stage I and II patients (3%) (26). Since relatively early-stage cancers are indicated for surgery, the impact of cancer on nutrition for those who undergo MCS is less than those in the late stages of cancer. Our study also indicated that subjects with relative early-stage cancer and PEM were more likely to be older, female, black, have low incomes, receive the operation in rural, urban non-teaching hospitals and lower-volume centers, and have more comorbidities, and were less likely on private insurance (**Table 1**). The difference in PEM rates among patients with different races, income statuses, properties and regions of hospitals, and types of insurance may be attributable to socioeconomic factors. Concerning female PEM patients, accumulating evidence suggests that vitamin disbalance play an important role in women’s health and nutraceutical supplementation is an effective way to improve the situation (27, 28). Our results highlight the importance of targeting such groups who are susceptible to malnutrition and may lack nutrition support.

As cancer-related malnutrition is still largely unidentified, underestimated, and undertreated in clinical practice, many screening tools have been recommended. Groups including the European Society for Clinical Nutrition and Metabolism and the American Cancer Society have been developing guidelines regarding nutrition in cancer patients (29, 30). Our study revealed that the prevalence of PEM among patients for MCS was continuously risen. As the importance of assessing nutritional status before cancer surgery has gained more notice by surgeons, there is reason to believe that the increasing prevalence of PEM is owing to improvements in its detection rate. Meanwhile, the mortality rate in both the PEM and non-PEM groups decreased from 2009 to 2015, and the EAPC of mortality was -4.52 and -4.21%, respectively, which implies the rate of mortality decrease seen in the PEM group exceeds that of the non-PEM group. Notably, other studies have also shown a decreasing trend in mortality after MCS from 1999 to 2009, with a reported EAPC of -2.4%. During the same period, the overall mortality in all patients undergoing MCS was 2% (31). This study extends this knowledge. Meanwhile, a declining EAPC of major complications is only seen in the PEM group (-1.21%, 95% CI [-1.85–0.56], P < 0.01). This suggests that improved methods for the identification, prevention, and treatment of malnutrition in cancer patients have already made some difference.

Despite great advances in surgical techniques, postoperative recovery of cancer patients is tortuous, where malnutrition plays a major role (32). Our nationwide data analysis revealed that patients with PEM had a 2.26-fold risk of mortality compared to those without PEM after MCS, which was consistent with previous studies focusing on one specific cancer. Data analysis based on American College of Surgeons-National Surgical Quality Improvement Program from 2009 to 2013 indicated that patients with mild hypoalbuminemia, an indicator for malnutrition, had significantly higher postoperative mortality rates of colorectal cancer than those with normal albumin levels (OR=1.74; P < 0.001) (33). Furthermore, we made subgroup analysis to explore the influence of surgical type on mortality of PEM patients. Noteworthily, PEM patients had significantly high risk of mortality when undergoing non-GI surgery, including prostatectomy, hysterectomy and lung resection (**Table 3**). It is reasonable that malnutrition is more common in patients with GI cancers due to GI side effects of nausea, vomiting, anorexia, diarrhea, dysphagia, and malabsorption (34). However, once PEM occurs in patients with non-GI cancers, it always means that the patient’s physical condition is very poor; therefore, the impact of PEM may be more pronounced in such cases. Besides, it is reported that prostate cancers and cancers involving uterine corpus are generally diagnosed at lower stages and grades. In contrast, esophageal cancer and pancreatic cancer are generally diagnosed at later stages and are related to lower survival rates (4), which might also partially explain the strong effects of PEM on prostatectomy and hysterectomy as well as its relatively weak effects on esophagectomy and pancreatectomy. This suggests more attention should be paid to non-GI cancer patients with PEM whose nutritional statuses are always less noticed than GI cancer patients. Urgent and appropriate nutritional supplements should be administered to patients with PEM, thereby correcting PEM and improving their prognosis.

Apart from mortality, major complications play the key roles in perioperative recovery, hospital stay and total cost of cancer patients (35). Our study indicated that patients with PEM have a 2.46-fold increased risk of overall major complications compared to those without PEM after MCS (**Table 2**). It is worth noting that the highest OR related to PEM was sepsis (OR=3.08) and septic shock (OR=3.55), which was consistent with previous report (1). Cancer patients are considered to have baseline immunosuppression (36), and PEM worsens this condition, which inclines patients to immunologic deficiency due to protein deficiency and lack of immune mediators and consequently predisposes patients to susceptibility to infection (37). Sepsis always results in massive catabolism, characterized by the depletion of protein, fat, and glycogen energy reserves. It is common for patients with sepsis to experience muscle wasting and weight loss, which further causes or worsens malnutrition (38). Therefore, early screening of PEM and monitoring for infection symptoms, signs, and laboratory findings are crucial for cancer patients undergoing surgery. Furthermore, there was a higher risk of pneumonia (OR=2.52), adult respiratory distress syndrome (2.51), and mechanical ventilation (OR=2.62) in patients with PEM after MCS, which were resulted mainly from PEM-induced muscle weakness and PEM-related immunologic deficiency (39, 40). Also, higher risk of cardiac complications (acute myocardial infarction, cardiac arrest) were also observed from our study, which may result from high levels of inflammation and the progression of atherosclerosis (41) as well as cardiac structural alterations and the occurrence of heart failure (42).

There are several limitations of our study. First, the use of ICD-9-CM codes to identify these procedures and events relies largely on coding accuracy, which could be assigned erroneously. As PEM has not been rigorously validated in the NIS, if the misclassification occurs, it is impossible to access individual patient charts to confirm the diagnosis, which inevitably results in bias. Second, the NIS data set does not provide information for tumor stage and grade, laboratory values, or other cancer-related treatment received by the patients, which made it impossible to evaluating these parameters on outcomes. Third, the NIS data does not provide consistent surgeon identification, and there is a possible relationship between outcomes after MCS and the experience and practice patterns of surgeons or institutions. Fourth, as the information after discharge is not available from the NIS, the post-discharge outcomes could not be evaluated. Fifth, since the heterogenous patients and the restrictions of NIS database, it is not possible to extrapolate the information for each single cancer surgery. Despite these shortcomings, the NIS is a large and reliable database containing hospitalized patient data from over 4,000 hospitals in over 30 states in the United States, and temporal trend analyses are performed during a 6-year time span, which affords more power to the study. Moreover, the database has been widely applied in other retrospective studies. In addition, the impact of PEM on outcomes is independent of confounding variables in the multivariable and double robust inverse probability of treatment weighting method. Also, we investigate the influence of surgical type on perioperative outcomes, aiming to provide more comprehensive information concerning surgical type and relating outcome. Since the present study was observational, prospective studies are needed to verify the impact of PEM on worse outcomes after MCS.

In conclusion, we found PEM had severe impact on mortality, major complications, total cost and LOS of cancer patients underwent MCS by analyzing data of more than one million patients. PEM patients who underwent lung resection and colectomy had the highest mortality rate while PEM significantly increased the risk of mortality and major complications in those underwent prostatectomy, hysterectomy and lung resection. Also, we discovered consistently increasing PEM rates and the conversely decreasing EAPC of both mortality and major complications in the PEM group undergoing MCS from 2009 to 2015, which are likely the result of improved screening tools, evolving guidelines, and better management. Prompt recognition of PEM and the initiation of appropriate nutrition therapy is essential to achieve better outcomes after MCS.

## Data availability statement

The original contributions presented in the study are included in the article/**Supplementary Material**. Further inquiries can be directed to the corresponding authors.

## Author contributions

JJ, XZ, ZD, YL, and HL designed research. JJ, XZ, PZ, and YX conducted research. JJ, ZD, and HH analyzed data. JJ, XZ, YL, and HL wrote the paper. All authors contributed to the article and approved the submitted version.
