# Supplementary material for: Protein-energy malnutrition and worse outcomes after major cancer surgery: A nationwide analysis
Source: Front Oncol. 2023 Jan 17;13:970187. doi: 10.3389/fonc.2023.970187 (PMC9886875; doi:10.3389/fonc.2023.970187)
Supplement: Supplementary file 1 [file Table_1.docx]

**Supplementary Table 1. Cases identification for the study**

| **Cancer surgical type** | **ICD-9-CM diagnostic codes** | **ICD-9-CM procedural codes** |
| --- | --- | --- |
| Colectomy | 457, 4571, 4572, 4573, 4574, 4575, 4576, 4579, 458, 4581, 4582, 4583 | 153.x |
| Cystectomy | 577, 5771, 5779 | 188.x |
| Esophagectomy | 424, 4240, 4241, 4242 | 150.x |
| Gastrectomy | 435, 436, 437, 439, 4391, 4399 | 151.x |
| Hysterectomy | 683, 6831, 684, 6841, 685, 6851, 687, 6871 | 182.x |
| Lung resection | 323, 3230, 3239, 324, 3241, 3249, 325, 3250, 3259 | 162.x |
| Pancreatectomy | 526, 527, 525, 5251, 5252, 5253, 5259 | 156.x, 157.x |
| Prostatectomy | 605 | 185.x |

**Supplementary Table 2. ICD-9-CM codes for PEM and complications**

| **Variables** | **ICD-9-CM diagnostic codes** |
| --- | --- |
| PEM | 260, 261, 262, 263, 2698, 7994, 7833, 7837, 78321, 78322 |
| Pneumonia | 481, 482, 4821, 4822, 4823, 48230, 48231, 48232, 48239, 48240, 48241, 48249, 48280, 48281, 48282, 48283, 48284, 48289, 48290, 4830, 4831, 4838, 485, 486, 4870, 9973, 5070 |
| Pulmonary embolism | 415 |
| Acute kidney injury | 584, 5845, 5846, 5847, 5848, 5849 |
| Acute ischemic stroke | 436, 4371, 43301, 43311, 43321, 43331, 43381, 43391, 43401, 43411, 43491 |
| [Acute](javascript:;) [myocardial](javascript:;) [infarction](javascript:;) | 41001, 41011, 41021, 41031, 41041, 41051, 41061, 41071, 41081, 41091 |
| Cardiac arrest | 4275 |
| Acute Respiratory Distress Syndrome | 51881, 51882, 51884 |
| Sepsis | 99591 |
| Septic Shock | 99592 |

**Supplementary Table 3. Distribution of Elixhauser comorbid conditions in patients who underwent major cancer surgery with and without PEM**

| **Conditions** | With PEM  (N=19201, %) | Without PEM  (N=249394, %) | P-value |
| --- | --- | --- | --- |
| Alcohol abuse | 868(4.52) | 4418(1.77) | <0.0001 |
| Acquired Immune Deficiency Syndrome | 35(0.18) | 149(0.06) | <0.0001 |
| Deficiency anemias | 6536(34.04) | 32299(12.95) | <0.0001 |
| Rheumatoid arthritis/collagen vascular diseases | 379(1.97) | 4150(1.66) | 0.0016 |
| Chronic blood loss anemia | 1324(6.90) | 5497(2.20) | <0.0001 |
| Congestive heart failure | 2205(11.48) | 9541(3.83) | <0.0001 |
| Chronic pulmonary disease | 4760(24.79) | 46231(18.54) | <0.0001 |
| Coagulopathy | 1669(8.69) | 5298(2.12) | <0.0001 |
| Depression | 1735(9.04) | 17236(6.91) | <0.0001 |
| Diabetes, uncomplicated | 3683(19.18) | 45754(18.35) | 0.0126 |
| Diabetes with chronic complications | 690(3.59) | 4701(1.88) | <0.0001 |
| Drug abuse | 268(1.40) | 1626(0.65) | <0.0001 |
| Hypertension, uncomplicated and complicated | 10512(54.75) | 140297(56.26) | 0.0008 |
| Hypothyroidism | 1934(10.07) | 22508(9.03) | <0.0001 |
| Liver disease | 609(3.17) | 3742(1.50) | <0.0001 |
| Lymphoma | 130(0.68) | 1213(0.49) | 0.0005 |
| Fluid and electrolyte disorders | 10361(53.96) | 35247(14.13) | <0.0001 |
| Obesity | 1884(9.81) | 32154(12.89) | <0.0001 |
| Other neurological disorders | 1107(5.77) | 7488(3.00) | <0.0001 |
| Paralysis | 363(1.89) | 1473(0.59) | <0.0001 |
| Peripheral vascular disorders | 1534(7.99) | 9968(4.00) | <0.0001 |
| Psychoses | 712(3.71) | 4293(1.72) | <0.0001 |
| Pulmonary circulation disorders | 1066(5.55) | 4019(1.61) | <0.0001 |
| Renal failure | 2006(10.45) | 11747(4.71) | <0.0001 |
| Peptic ulcer disease excluding bleeding | 45(0.23) | 100(0.04) | <0.0001 |
| Valvular disease | 1038(5.41) | 8374(3.36) | <0.0001 |
| Weight loss | 18370(95.67) | 29(0.01) | <0.0001 |

PEM, protein-energy malnutrition.

**Supplementary Table 4. Comparisons of clinical outcomes in patients with and without PEM according to cancer surgical type**

| **Outcomes** | **Mortality (%)** | | **Major complications** | | **Total cost, median ($)** | | **LOS, median (days)** | |
| --- | --- | --- | --- | --- | --- | --- | --- | --- |
|  | **PEM** | **No PEM** | **PEM** | **No PEM** | **PEM** | **No PEM** | **PEM** | **No PEM** |
| Colectomy | 8.35 | 2.59 | 47.14 | 18.03 | 33957 | 18731 | 13 | 7 |
| Cystectomy | 4.64 | 1.29 | 55.19 | 18.91 | 44258 | 27836 | 15 | 8 |
| Esophagectomy | 7.13 | 3.79 | 51.01 | 29.23 | 55995 | 41354 | 15 | 10 |
| Gastrectomy | 7.38 | 2.78 | 43.48 | 20.25 | 41150 | 28128 | 14 | 9 |
| Hysterectomy | 5.87 | 0.26 | 41.29 | 5.91 | 28506 | 12120 | 10 | 3 |
| Lung resection | 10.27 | 1.62 | 51.26 | 16.50 | 37329 | 21422 | 14 | 6 |
| Pancreatectomy | 5.99 | 2.72 | 35.65 | 15.84 | 44161 | 30627 | 15 | 9 |
| Prostatectomy | 1.60 | 0.04 | 32.45 | 2.02 | 23232 | 12132 | 5 | 1 |

PEM, protein-energy malnutrition; LOS, length of stay.

**Supplementary Table 5. Sensitivity analysis of PEM and clinical outcomes in patients undergoing major cancer surgery based on double robust inverse probability of treatment weighting method**

| **Outcomes** | **Adjusted OR (95%CI)** | **P-value** |
| --- | --- | --- |
|  |  |  |
| Mortality | 3.71(3.05,4.51) | <0.0001 |
| Major complications | 3.73(3.33, 4.19) | <0.0001 |
| Pneumonia | 3.83(3.26,4.50) | <0.0001 |
| Pulmonary embolism | 2.33(1.89,2.87) | <0.0001 |
| Renal failure | 3.38(2.93,3.91) | <0.0001 |
| Acute ischemic stroke | 2.71(2.07,3.55) | <0.0001 |
| Acute myocardial infarction | 1.84(1.45,2.34) | <0.0001 |
| Cardiac arrest | 2.43(1.92,3.07) | <0.0001 |
| Adult respiratory distress syndrome | 3.81(3.15,4.62) | <0.0001 |
| Sepsis | 4.47(3.54,5.63) | <0.0001 |
| Septic shock | 7.12(5.38,9.40) | <0.0001 |
| Mechanical Ventilation | 3.95(3.35,4.65) | <0.0001 |
| Total cost (coefficient) | 0.44 | <0.0001 |
| Length of stay (coefficient) | 0.63 | <0.0001 |

PEM, protein-energy malnutrition; OR, odds ratio; CI, confidence interval.
